# Magnetic Resonance Image findings of Spinal Tuberclosis at first presentation

**DOI:** 10.1186/1755-7682-7-12

**Published:** 2014-03-22

**Authors:** Arsalan Ahmad Alvi, Aisha Raees, Muhammad Asim khan Rehmani, Hafiz Muhammad Aslam, Shafaq Saleem, Junaid Ashraf

**Affiliations:** 1 Dow Medical College, Dow University of Health Sciences, Karachi, Pakistan; 2 Assistant Professor, Department of Neurosurgery at Civil Hospital, Dow University of Health Sciences, Karachi, Pakistan; 3 Professor of Neurosurgery, Department of Neurosurgery at Civil Hospital, Principal Dow Medical College, Dow University of Health Sciences, Karachi, Pakistan

**Keywords:** Tuberculosis, Paraplegia, Lumbar

## Abstract

**Background:**

Spinal tuberculosis presents in various pathological patterns. The clinical presentation and often the management depend on exact pathological findings. Objective of study was to evaluate the Pathology of spinal tuberculosis as depicted by MRI findings in 119 consecutive cases of spinal TB.

**Methodology:**

It was a cross sectional and observational study conducted at Civil Hospital, Karachi from July 2010 to December 2012.Total numbers of participants were 119. Diagnosis was based on positive histopathology results along with the supportive evidence in MRI. A pre-structured questionnaire was constructed to record the data. Study was ethically approved by Institutional Review Board of Dow University of Health Sciences. Sample size was calculated by using Open-EPI software. All the data was entered and analyzed through SPSS 19.

**Result:**

There were 119 patients who participated in this study out of which 52 were males and 67 were females. Most common level was Dorso-lumbar (33.6%) and 87.5% of them had spondylodiscitis while 90% had cord compression. All 6 (100%) patients who had their upper- dorsal region affected had gibbus formation while all those patients having lumbosacral region involved had thecal compression 4 (100%). Most common mode of treatment used in patients having Spinal TB at Lumbar region was conservative (86.2%).

**Conclusion:**

MRI findings were mostly shadowed with features such as disc destruction and thecal or cord compression. MRI scan could be used for early detection of spinal TB which can reduce disability and deaths in patients. Major clinical findings in spinal TB were fever, Para paresis and back pain.

## Introduction

Since times in memorial tuberculosis has been an infectious disease to affect millions of people around the world. A bacteria known as mycobacterium tuberculosis is a culprit for causing tuberculosis [1]. Pakistan is one of those countries who have the highest burden of TB patients [2,3]. According to WHO, Spinal TB ranked eighth in the world Extra-Pulmonary TB [4]. This disease is showing resurgence in the developed countries while in the developing part of the world it’s showing an alarming increase in the number of sufferers [5]. Spinal TB is also known as Pott’s disease. It accounts for less than 1% of all cases of TB [6].

Two distinct types of spinal TB can be identified, the classic form, called spondylodiscitis (SPD), and a more common atypical form characterized by spondylitis without disk involvement (SPwD) [7]. Tuberculosis usually affects the lung before spreading to the spine through the batson’s venous plexus or by lymphatic drainage [8].The spinal TB commonly spread into the intervertebral disc through neighboring vertebra while in children it spreads due to vascularized nature of intervertebral disc and it is the anterior region of the vertebra body that is most commonly affected [9,10]. This infection poses a difficult task in the initial stages for diagnosis before Kyphotic deformity or neurological deficit takes place. Tuberculosis remains a leading cause of morbidity and mortality worldwide. Skeletal TB makes up 20 percent of extra pulmonary TB. It is also worth noting that 50 to 60 percent of this skeletal TB has an impact on spine and if there is a delay in diagnosis and management of this dreadful disease it can lead to spinal cord compression and spinal deformity [11,12]. This disease spread to the vertebra through the blood and it mostly affect the anterior subchondral region of vertebral body which can directly extent to the neighboring intervertebral disc [13]. It can also lead to the involvement of soft tissue and cause abscess and in worse case it can give rise to gibbus deformity and neurological complication [13]. As spinal TB is a major cause of non-traumatic paraplegia in under developed countries like Pakistan [14], it was felt necessary to evaluate and observe the pathological findings of Spinal TB on MRI.

## Methodology

### Study settings

It was a cross sectional and observational study conducted in the Civil Hospital Karachi, affiliated with Dow University of Health Sciences. Study was conducted from July 2010 to December 2012.

### Study participants

Study was conducted on the diagnosed patients of Spinal TB therefore convenient sampling was taken. Children under the age of five years and people who did not meet our diagnostic criteria were excluded from our study.

### Diagnostic criteria

#### Either

• Positive result of biopsy

OR

• Culture from abscess positive to acid fast stain

OR

• Respond to anti tuberculosis drug according to WHO regimen with three months follow up with MRI. **For the purpose of this study we used the MRI scan when patient first presented to us. The MRI that was taken afterwards was only used for the purpose of diagnosis and how well the patients were responding to treatment.**

### Supportive diagnostic criteria

• MRI and CT scan were not used as diagnostic modality as they are not specific for tuberculosis. They are able to show amount of damage to vertebra and MRI is able to show the spread of disease and neurological deficit, but above criteria are more accurately able to diagnose this disease [15].

### Medical

The treatments that were given to the patient met the WHO criteria. The treatment was divided into two phase, in the first phase which also known as an intensive phase **in which** a combination of isoniazid, rifampicin, streptomycin, and pyrazinamide **were given** for two months, followed by the second phase known as continuous phase in which patients were offered the combination of isoniazid and rifampicin. Due to the risk of complication like disability and mortality, WHO recommends 9 months treatment [9,16].

### Surgery

Those patients who after one month of medical treatment still showed a power grade 0 or worse neurological condition were surgically treated. Anterior decompression and stabilization was the surgical procedure of choice.

### Consent

Before entry into the study, subjects were informed about the aims and methods. Subjects will be informed that their participation is voluntary. They will be informed that choosing not to participate will not affect their care. After giving sufficient information written consent was obtained and confidentiality of data was maintained.

### Ethical review

Study was approved ethically by the Institutional Review Board of Dow University of Health Sciences.

### Analysis

All the analysis for this research was done by using SPSS 19. Sample size was calculated by using Open-EPI software. Mean and Standard deviation were evaluated for continuous data and for categorical data frequency and percentage were calculated. Correlations among variables were explored by using Spearman rank test, Threshold of significance was set at 0.05.

## Result

There were total numbers of 119 participants who met our diagnostic criteria and were willingly to take part in this study. The level of spine which was most commonly found to be affected was the Dorsa lumbar (D10 to D12 or D12 with L1) {N = 40 (33.6%)}. In this study relationship between MRI finding and level of spine affected by T.B was observed (Table 1). In cervical level classical form of T.B was present in 11 (57.9%) and also the highest number of patients had involvement of anterior region of vertebra in this level which was 16 (84.2%) but in upper dorsal level it was majorly involvement of all three region anterior, posterior, and lateral region altogether which was 2 patients (33.3%). Kyphotic angle and soft tissue involvement was seen in all 6 (100%) participants who had complaint in the upper dorsal area. All 6 patients (100 percent) who had their upper dorsal level infection had gibbus deformation. Cord compression was seen in 36 (90%) of people presenting with TB in their dorsa lumbar region and all the patients with lumbar and lumbosacral had thecal compression with a frequency of 29 (100%), and 4 (100%) respectively. (Table 2). In terms of percentage of vertebral body destruction, 6 (31.6%) patients had their cervical level affected less than 25% whereas 4 out of 6 (66.7%) of upper dorsal patient had more than 75% vertebra body destruction (Table 3). There were 16 (84.2%) patients with both epidural and paravertebral abscess in cervical region (Table 4). In this study the relationship between level of spine and symptomatology was also observed (Table 5). In this part of the research the values were found to be significant p-value (<0.05) for Para paresis, Quadra paresis, back pain, neck pain, and quadriplegia. Quadra paresis was seen in people who only had their cervical region involve with the frequency of 10 (52.6%), while quadriplegia was seen in 4 (21.1%) patients with cervical level involvement. Bowel or bladder dysfunction was found to be common complaint in patient who had their upper dorsal spine involved with 4 out of 6 (66.7%) facing this problem. It was observed that the highest number of Para paresis were found in patient with the complaint presenting in dorsa lumbar region with 28 out of 40 patients (70%) stating they have weakness and on examination and they were found to have decreased power. It is also worth noting that all the patients who had their lumbosacral area involved 4 (100%) were also found to have a fever and back pain.

**Table 1:** Relationship between level of spine and MRI finding

**Level**	**Cervical**	**Upper Dorsal**	**Mid-Dorsal**	**Dorsa lumbar**	**Lumbar**	**Lumbosacral**	**P-Value**
	**N = 19**	**N = 6**	**N = 21**	**N = 40**	**N = 29**	**N = 4**	
Gibbus	3	6	12	26	13	1	N.S
	15.8%	100.0%	57.1%	65.0%	44.8%	25.0%	
Spondylodiscitis	11	5	18	35	25	4	N.S
(classic form)	57.9%	83.3%	85.7%	87.5%	86.2%	100.0%	
Spondylitis without disc involvement (atypical form	8	1	3	5	4	0	N.S
	42.1%	16.7%	14.3%	12.5%	13.8%	0%	
Cord compression	17	5	18	36	0	0	<0.001
	89.5%	83.3%	85.7%	90.0%	.0%	.0%	
Thecal compression	0	0	0	4	27	4	<0.001
	.0%	.0%	.0%	10.0%	93.1%	100.0%	
Hypnotic angle	7	6	16	30	18	2	N.S
	36.8%	100.0%	76.2%	75.0%	62.1%	50.0%	
Soft tissue	18	6	20	37	26	3	N.S
Involvement	94.7%	100.0%	95.2%	92.5%	89.7%	75.0%	

**Table 2:** Relationship between level of spine and vertebral body

**Level**	**Cervical**	**Upper Dorsal**	**Mid-Dorsal**	**Dorsa lumbar**	**Lumbar**	**Lumbosacral**	**P-Value**
	**N = 19**	**N = 6**	**N = 21**	**N = 40**	**N = 29**	**N = 4**	
Anterior region of vertebral body	16	1	10	21	21	3	NS
	84.2%	16.7%	47.6%	52.5%	72.4%	75.0%	
Posterior region of vertebral body	0	0	0	0	0	0	NS
	0%	0%	0%	0%	0%	0%	
Lateral region of vertebral body	0	1	0	0	0	0	NS
	0%	16.7%	0%	0%	0%	0%	
Anterior and posterior region of vertebral body	0	1	1	2	2	0	NS
	0%	16.7%	4.8%	5.0%	6.9%	0%	
Anterior and lateral region of vertebral body	3	1	5	7	4	0	NS
	15.8%	16.7%	23.8%	17.5%	13.8%	0%	
All region involved of vertebral body	0	2	5	10	2	1	NS
	0%	33.3%	23.8%	25.0%	6.9%	25.0%	

**Table 3:** Relationship between level of spine and destruction of vertebral body

**Level**	**Cervical**	**Upper Dorsal**	**Mid-Dorsal**	**Dorsa lumbar**	**Lumbar**	**Lumbosacral**	**P-Value**
	**N = 19**	**N = 6**	**N = 21**	**N = 40**	**N = 29**	**N = 4**	
Less than 25% vertebra body destruction	6	0	4	7	4	0	NS
	31.6%	0%	19.0%	17.5%	13.8%	0%	
25% to 50% vertebra body destruction	4	1	5	9	6	1	NS
	21.1%	16.7%	23.8%	22.5%	20.7%	25.0%	
50% to 75% vertebra body destruction	4	1	7	11	12	2	NS
	21.1%	16.7%	33.3%	27.5%	41.4%	50.0%	
More than 75% vertebra body destruction	4	4	4	13	7	1	NS
	21.1%	66.7%	19.0%	32.5%	24.1%	25.0%	
No destruction vertebra body	1	0	1	0	0	0	NS
	5.3%	0%	4.8%	0%	0%	0%	

**Table 4:** Relationship between level of spine and abscess

**Level**	**Cervical**	**Upper Dorsal**	**Mid-Dorsal**	**Dorsa lumbar**	**Lumbar**	**Lumbosacral**	**P-Value**
	**N = 19**	**N = 6**	**N = 21**	**N = 40**	**N = 29**	**N = 4**	
Epidural abscess	0	0	5	8	4	1	NS
	0%	0%	23.8%	20.0%	13.8%	25.0%	
Paravertebral abscess	1	1	3	5	4	0	NS
	5.3%	16.7%	14.3%	12.5%	13.8%	.0%	
Epidural + Paravertebral abscess	16	5	10	21	18	3	NS
	84.2%	83.3%	47.6%	52.5%	62.1%	75.0%	
No abscess	2	0	3	6	3	0	NS
	10.5%	.0%	14.3%	15.0%	10.3%	0%	

**Table 5:** Relationship between level of spine and symptom logy

**Level**	**Cervical**	**Upper Dorsal**	**Mid-Dorsal**	**Dorsa Lumbar**	**Lumbar**	**Lumbosacral**	**P-Value**
	**N = 19**	**N = 6**	**N = 21**	**N = 40**	**N = 29**	**N = 4**	
Para paresis	1	4	14	28	18	3	0.02
	(5.3%)	(66.7%)	(66.7%)	(70%)	(62.1%)	(75.0%)	
Quadra paresis	10	0	0	0	0	0	<0.001
	(52.6%)	(0%)	(0%)	(0%)	(0%)	(0%)	
Back pain	2	6	21	40	28	4	<0.001
	(10.5%)	(100.0%)	(100.0%)	(100.0%)	(96.6%)	(100%)	
Neck pain	18	3	0	1	0	0	<0.001
	(94.7%)	(50.0%)	(0%)	(2.5%)	(0%)	(0%)	
Bowel or bladder dysfunction	6	4	11	16	7	2	N.S
	(31.6%)	(66.7%)	(52.4%)	(40.0%)	(24.1%)	(50.0%)	
Sensory disturbance	8	0	12	21	7	2	N.S
	(42.1%)	(0%)	(57.1%)	(52.5%)	(24.1%)	(50.0%)	
Paraplegia	2	2	6	9	4	1	N.S
	10.5%	33.3%	28.6%	22.5%	13.8%	25.0%	
Quadriplegia	4	0	0	1	0	0	0.008
	21.1%	.0%	.0%	2.5%	.0%	.0%	
Fever	13	4	15	28	21	4	N.S
	68.4%	66.7%	71.4%	70.0%	72.4%	100.0%	
Malaise	13	4	15	28	19	4	N.S
	68.4%	66.7%	71.4%	70.0%	65.5%	100.0%	

## Discussion

Objective of the study was to evaluate the pathology of Spinal T.B as depicted by MRI findings at first presentation. Since MRI was the earliest diagnosis modality through which patients of spinal T.B can be picked because of specific structural changes in the spine of soft tissue and disc, but the confirmatory diagnosis could only be made on biopsy or culture [17]. So the only purpose the MRI was used to see the pathological changes occurring in the spine due to this illness in the patient. On the MRI scan most common region found to be affected was dorsal lumbar 33.3% which was also seen in previous studies [5]. The greatest number of patients that were encounter belonged to female who had the age between 11 to 19 years on the other hand it was the male that dominant the outcomes in prior research [5]. In this study mid dorsal region was seen commonly associated with urinary incontinence, while a research conducted in India also showed the same result [18]. Back pain remained the most frequently recorded symptom and fever was found to be more regular finding than developed country as these symptoms mostly compel the patient in our setting to pay a visit to doctor [19,20]. Kyphosis was found to be presenting more commonly than previously thought while Para paresis was seen to be as common as previously presumed which shows that there was a delay in getting proper treatment [21-23]. Another study showed that kyphosis was found to be a valuable marker for spinal tuberculosis and this study also showed that majority of patients had some disturbance on the angle of spine [24]. MRI also showed that both epidural and Para vertebral abscess exited together in most of the patients who had shown abscess formation likewise also shown in earlier studies, but the number of people with cord compression was more occasional outcome in our situation [17,25,26]. Paravertebral abscess was a less common finding compared to study conducted in southwest china while overall there were more number of patients presenting with other type of abscess which shows that majority of people presenting to this setup were delay in seeking treatment for this disease [23]. Disc destruction (spondylodiscitis) was similar to study conducted in India in 2012 while gibbus deformity was significantly higher which is understandable because they have stated that they rarely conduct contrast enhanced MRI scans due to being expensive hence it shows without this scan many underlying deformity would be missed which can be a source of pain or disability [27]. The fascinating feature of this research was that all participants who had lumbosacral region involved on MRI also have thecal compression. In contrast to other studies which showed anterior and posterior vertebral body destruction together, our finding showed anterior to be most commonly implicated [28]. The debris, pus and granulation from the bone destruction leads to one of the most dreaded complication of this disease known as paraplegia although pus formation was a common finding in this study but surprisingly paraplegia was not that common [29]. The patient who had their upper dorsal region affected were more likely to have severe body destruction than any other region, while people who had cervical region affected were less expected to undergo severe vertebral body destruction. In all the patients, the ESR was highly raised and as the treatment went on, ESR was shown to be decreased. The findings of this study were comparable with past study [30]. For conservative treatment the WHO regimen of anti-tuberculosis drug was followed and the follow up MRI was taken after three months, to observe the affectedness of treatment. All those patients who had neurological deficits after one month of conservative treatment underwent anterior decompression and stabilization like the study conducted in Minnesota [28]. After the healing of spinal tuberculosis there are chances of deformity occurring leading to neurological deficits which had the worse prognosis and this can be only maintained if patients were kept on a follow up and monitoring through MRI even after the disease had been treated [9,31].

There were number of reasons for the delay in consultation like majority of patients belong to poor socioeconomic stratum, lack of education, and most of these patients were coming from rural areas of Sindh which lacks health infrastructure. This delay results in great damage to spine which can be observe clearly on the MRI scan [32]. If patients were compel to seek early attention from doctors or create awareness about the symptoms than there would be less deformities c.

### Limitation

This study was not free from limitations. The most important limitation was that it was conducted at just one tertiary care hospital of Karachi, which does not allow us to predict the overall situation in whole country. Furthermore, convenient sampling was employed, which may have led to selection bias, and hence is not truly representative of the population under study. There is always room for improvement and we would encourage more people to conduct research related to that.

### Future studies

This research opens the door of large proportion of research which needs to be done on this topic to define more accurately the true burden of Spinal tuberculosis in developing countries. It’s also necessary to do large scale trials on the implication of pathological changes with reference to tuberculosis treatment.

## Conclusion

It was concluded that dorsa lumbar region was the mostly affected region in this disease and the female teenagers were majorly associated with it. Commonest symptoms shown were back pain, Para paresis, and fever. MRI scans showed soft tissue involvement, cord compression, and disc destruction were major findings to be looked over in lumbosacral region and in lumbar spine, familiar observation was thecal compression which could be prevented by giving early decompression. The treatment mostly applied was conservative but in some cases surgical intervention was also required.

## Abbreviations

T.B: Stands for Tuberculosis; N.S: Stands for not significant.

## Competing interest

Authors declared that they had no competing interest.

## Authors’ contribution

AAA, AR and HMA did manuscript drafting and analyzing. SS did data collection and data entry into SPSS. MAKR and JA did review critically and also supervise the project. All authors check and approve the final version.
